# Dopamine manipulations modulate paranoid social inferences in healthy people

**DOI:** 10.1038/s41398-020-00912-4

**Published:** 2020-07-05

**Authors:** J. M. Barnby, V. Bell, Q. Deeley, M. A. Mehta

**Affiliations:** 1 Social and Cultural Neuroscience Research Group, Centre for Neuroimaging Sciences, Institute of Psychiatry, Psychology, and Neuroscience, King’s College London, London, UK; 2 Research Department of Clinical, Educational, and Healthy Psychology, University College London, London, UK; 3 Social and Cultural Neuroscience Research Group, Forensic and Neurodevelopmental Sciences, Institute of Psychiatry, Psychology, and Neuroscience, King’s College London, London, UK

**Keywords:** Human behaviour, Neuroscience

## Abstract

Altered dopamine transmission is thought to influence the formation of persecutory delusions. However, despite extensive evidence from clinical studies there is little experimental evidence on how modulating the dopamine system changes social attributions related to paranoia, and the salience of beliefs more generally. Twenty seven healthy male participants received 150mg L-DOPA, 3 mg haloperidol, or placebo in a double-blind, randomised, placebo-controlled study, over three within-subject sessions. Participants completed a multi-round Dictator Game modified to measure social attributions, and a measure of belief salience spanning themes of politics, religion, science, morality, and the paranormal. We preregistered predictions that altering dopamine function would affect (i) attributions of harmful intent and (ii) salience of paranormal beliefs. As predicted, haloperidol reduced attributions of harmful intent across all conditions compared to placebo. L-DOPA reduced attributions of harmful intent in fair conditions compared to placebo. Unexpectedly, haloperidol increased attributions of self-interest about opponents’ decisions. There was no change in belief salience within any theme. These results could not be explained by scepticism or subjective mood. Our findings demonstrate the selective involvement of dopamine in social inferences related to paranoia in healthy individuals.

## Introduction

Paranoia involves unfounded beliefs that others intend harm^1^. Epidemiological evidence suggests that paranoia exists on a spectrum in the general population, ranging from mild social concerns to persecutory delusions^2,3^. Observational and experimental research has identified a range of personal and interpersonal factors that influence paranoia. On the personal level, worry^4^, insomnia^5^, belief inflexibility^6^, and safety behaviours^7^ all contribute to the formation and/or maintenance of paranoia. In terms of social factors, social disadvantage and victimisation^8^, trauma^9^, and poor social support^10^ all play a role.

Neurobiologically, the subcortical dopamine system has been cited as a candidate for a ‘final common pathway’ on which accumulated biological, psychological and social stresses might have their most significant impact leading to the symptoms of psychosis^11,12^ of which persecutory delusions are the most common symptom^13^. Although the status of subcortical dopamine as a common pathway has been debated^14^, there remains extensive evidence for the dysregulation of the subcortical dopamine system in psychosis and the paranoia spectrum. Observational PET neuroimaging has found increased striatal dopamine in people at high-risk of progression to psychosis^15,16^, as well as prior to^16^ and during^17^ episodes of psychosis. Antipsychotic medication primarily has its effect through antagonism at D_2_ dopamine receptors in the mesolimbic and nigrostriatal pathway^18^. Additionally, stimulant drugs, which increase activity at mesolimbic D_2_ dopamine receptors, raise the risk of psychosis—with over 40% of recreational methamphetamine users developing psychosis^19^ of which paranoid delusions are the dominant symptom^20^.

The mechanisms that connect dysregulated dopamine to the symptoms of psychosis have been much debated. Several theories have suggested that striatal dopamine is involved in a process of aberrant salience attribution whereby meaningful connections are made between unrelated events or information, which form the basis for delusional beliefs^18,21,22^. This has been interpreted in terms of the neuromodulatory effect of dopamine on the integration of prediction error in hierarchical Bayesian models of perceptual learning^23,24^. In these models it has been proposed that altered dopamine transmission leads to abnormally strong weighting of perceptual prediction errors that disrupts learning and eventually manifests as delusions. More specifically, recent computational modelling^25^ and integrative socio-developmental cognitive accounts^12^ have suggested that disruption to dopamine-mediated processes underlying social interaction may be an important explanatory factor in persecutory delusions.

The evidence base for current theories of delusion largely rely on clinical studies, and there are far fewer studies that have taken the additional step of experimentally altering dopamine function in healthy participants to look for causal effects on psychosis-congruent beliefs. Studies have tested the effect of manipulating the dopamine system on the valuation of harm to others^26^, self-interest in economic decision-making^27^ and learning about others’ prosociality^28^. As far as we are aware, no studies to date have tested the effect of altering dopamine function on attributions of others’ intent to harm, the core social attributional process of paranoia^1^. Similarly, of the few existing pharmacological studies on delusion-related belief mechanisms, Krummenacher et al.^29^ found the effect of levodopa on perceptual sensitivity differed depending on levels of paranormal belief, chosen as a nonclinical analogue of delusional ideation. Mohr et al.^30^, also using levodopa, found that laterality of lexical decision processing altered as a function of magical ideation. However, belief salience^31^ has yet to be tested.

Given the importance of experimental pharmacological intervention studies to understand the mechanisms of psychopathology^32^, this study extends this work by examining how modulating dopamine affects (i) attributions of harmful intent—a core interpersonal process of paranoia; and (ii) salience of paranormal belief—chosen as a nonclinical analogue of delusional ideation and measured alongside salience of other beliefs. Healthy participants took part in a double-blind, within-subjects, randomised placebo-controlled trial of two drugs that alter the dopamine system –L-3,4-dihydroxyphenylalanine (levodopa or L-DOPA) to potentiate presynaptic dopamine, and haloperidol, to primarily block postsynaptic dopamine transmission via D_2_ receptors. At each stage, participants completed a game theoretic social inference task (multi-round Dictator Game; ref. ^33^) where participants were required to attribute the intentions of their partner after their partner had made a monetary decisions, and a measure of belief salience, that included paranormal beliefs^31^.

Given the role of dopamine in paranoia and paranoid delusions, we predicted that haloperidol would reduce attributions of harmful intent and salience of paranormal beliefs based on the observation that dopamine antagonism is the primary therapeutic mechanism of antipsychotics in the treatment of psychosis^34^. We predicted that potentiation of dopamine transmission using L-DOPA in healthy participants would increase attributions of harmful intent and the salience of paranormal beliefs, given increased presynaptic dopamine in those at risk of psychosis^11^. Following Barnby et al.^33^, we also predicted that haloperidol and L-DOPA would, respectively, reduce and increase the amount of trials taken to reach a peak level of high harmful intent attribution but not self-interest attributions. All analysis scripts and open data are available on the Open Science Framework (https://osf.io/mr63j/).

## Results

The study (Clinical Trials.gov Identifier: NCT03754062) also included the Salience Attribution Task^35^, although data from this task is not reported here. We preregistered the hypotheses and analysis for the multi-round Dictator Game (https://aspredicted.org/6zg2w.pdf) and belief salience measures (https://aspredicted.org/fh495.pdf) prior to unblinding.

We recruited 30 participants in total for the full experimental procedure and kept 27 for analysis. Two participants were removed from the analysis for having incomplete session data. One participant was removed from the analysis for having a very high Green et al Paranoid Thoughts Scale (GPTS; ref. ^36^) score (104) over two standard deviations away from the mean of the rest of the sample (46.52), potentially making our analysis less conservative.

### Demographics and baseline psychometrics

At baseline individuals recorded their age, ethnicity, political orientation, and filled out the Big-5 personality questionnaire^37^, brief OLIFE schizotypy questionnaire^38^, Bond and Lader mood rating scale^39^ for each drug condition pre and post dosing, and the Green Paranoid Thoughts Scale. Table 1 describes the distribution of these measures across the sample. Heart rate and blood pressure of participants at baseline and each study day are presented in Table 2.

**Table 1:** Age, mood ratings, and psychometrics of the included sample (*n* = 27).

Variable	Mean	SD	Min	Max	Range	Skew	Kurtosis	S.E.
GPTS	46.52	12.66	32	69	37	0.69	−0.93	2.44
Social reference	27.96	10.11	16	51	35	0.69	−0.72	1.95
Persecutory	18.56	3.60	16	31	15	1.85	3.23	0.69
OLIFE UE	2.41	2.27	0	7	7	0.49	−0.96	0.44
OLIFE CD	3.22	2.86	0	11	11	0.98	0.27	0.55
OLIFE IA	1.48	2.06	0	10	10	2.68	8.16	0.40
OLIFE IN	1.96	1.34	0	6	6	1.07	1.05	0.26
OLIFE Total	9.07	5.97	1	21	20	0.67	−0.72	1.15
Openness	40.07	5.73	25	49	24	−0.65	−0.32	1.10
Conscientiousness	34.74	3.03	28	40	12	−0.30	−0.74	0.58
Extraversion	30.26	3.68	25	37	12	0.07	−1.23	0.71
Agreeableness	35.15	3.52	29	43	14	0.52	−0.33	0.68
Neuroticism	24.70	3.12	20	32	12	0.47	−0.38	0.60
Age	29.44	8.69	20	52	32	1.23	0.72	1.67

Bond and lader mood rating scale								
	Alertness				Tranquil			
	Mean	SD	Min	Max	Mean	SD	Min	Max
*PLACEBO*								
VAS (pre)	43.07	2.40	10	90	36.00	3.49	7	64
VAS (post)	43.93	3.53	10	90	35.57	3.16	7	64
*L-DOPA*								
VAS (pre)	43.59	2.43	10	90	36.34	2.54	7	64
VAS (post)	43.21	2.57	10	90	35.79	2.02	7	64
*HALOPERIDOL*								
VAS (pre)	43.00	2.83	10	90	36.03	2.86	7	64
VAS (post)	44.21	3.82	10	90	36.00	1.96	7	64

Only the Bond and Lader scale (Visual Analogue Scale; VAS) was administered at baseline and subsequent study days, both before and after dosing.

*OLIFE* Oxford-liverpool inventory of feelings and experiences (Mason & Claridge, 2006), *UE* unusual experiences subscale, *CD* cognitive disorganisation subscale, *IA* introvertive anhedonia subscale, *IN* impulsive non-conformity subscale.

**Table 2:** Heart rate and blood pressure of participants at baseline and each study day.

Condition	Systole	Diastole	Heart rate	Systole	Diastole	Heart rate	Heart rate P-val.
BASELINE							
Mean	123.93	71.93	66.32				
SD	9.74	9.66	10.73				
	PREDOSE			POSTDOSE			
PLACEBO							
Mean	121.39	70.82	67.86	116.25	69.75	62.14	0.11
SD	8.71	9.83	8.19	24.89	17.49	16.11	
L-DOPA							
Mean	120.82	68.89	66.61	119.46	68.71	63.61	0.32
SD	9.66	7.92	10.62	9.12	9.34	9.80	
HALOPERIDOL							
Mean	121.36	69.54	67.71	116.36	67.61	60.75	<0.001
SD	9.78	7.95	10.03	9.68	7.77	10.09	
P-val.	0.21	0.36	0.72	0.012	0.07	0.2	

Formula for differences between sessions are “lmer (Systole/Diastole/HeartRate) ~ Drug Session + (1|ID)”. Paired *t*-tests were run for within-session heart rate.

### Multi-round Dictator game prediction 1: dopamine manipulation will moderate harmful intent attributions but not self-interest attributions

The multi-round Dictator game was a modified version of the dictator game, where participants were passive receivers of either unfair (100:0) or fair (50:50) splits of money over six trials with three different partners and were only required to infer the intentions of a partner, following each decision, down two dimensions: harmful intent or self interest on a scale of 0–100. While dictators were preprogrammed to either to unfair (always take the money), fair (always split the money), or partially fair (split the money half the time), unlike reinforcement learning paradigms, participants were not required to guess the type of Dictator they were matched with, and their attributions did not affect their monetary outcomes in subsequent trials. More details can be found in the methods. In placebo conditions, harmful intent and self interest attributions were not correlated with eachother overall or in each Dictator condition (*p*’s > 0.05).

We conducted three preregistered analyses for each dictator type. All reported statistics are beta coefficients of the top model following model averaging unless otherwise stated. See the Supplementary Materials for the mean values collapsed across conditions for harmful intent and self-interest attributions for each drug.

For unfair dictators, compared to placebo, haloperidol reduced harmful intent attributions (−1.155, 95% CI: −1.467, −0.845) but L-DOPA did not (−0.118, 95% CI: −0.410, 0.169). Compared to haloperidol, L-DOPA increased harmful intent attributions (1.037, 95% CI: 0.736, 1.348). Compared to placebo, haloperidol also increased self-interest attributions (0.650, 95% CI: 0.649, 0.651), but L-DOPA reduced self-interest attributions (−0.021, 95% CI: −0.022, −0.020). Compared to haloperidol, L-DOPA reduced self-interest attributions (−0.670, 95% CI: −0.671, −0.670).

For partially fair dictators, compared to placebo, haloperidol reduced harmful intent attributions (−0.420, 95% CI: −0.707, −0.133), but L-DOPA did not (0.169, 95% CI: −0.109, 0.446). Compared to haloperidol, L-DOPA increased harmful intent attributions (0.589, 95% CI: 0.303, 0.874). Compared to placebo, haloperidol also increased self-interest attributions (0.610, 95% CI: 0.362, 0.858) but L-DOPA did not (−0.054, 95% CI: −0.297, 0.188). Compared to haloperidol, L-DOPA reduced self-interest attributions (−0.665, 95% CI: −0.913, −0.416).

For fair dictators, compared to placebo, haloperidol reduced harmful intent attributions (−1.202, 95% CI: −1.202, −1.201), as did L-DOPA (−1.033, 95% CI: −1.034, −1.033). Compared to haloperidol, L-DOPA did not increase harmful intent attributions (0.167, 95% CI: −0.227, 0.561). Compared to placebo, haloperidol did not affect self-interest attributions (0.194, 95% CI: −0.078, 0.469), but L-DOPA decreased them (−0.331, 95% CI: −0.591, −0.070). Compared to haloperidol, L-DOPA reduced self-interest attributions (−0.526, 95% CI: −0.800, −0.254).

**Fig. 1:**
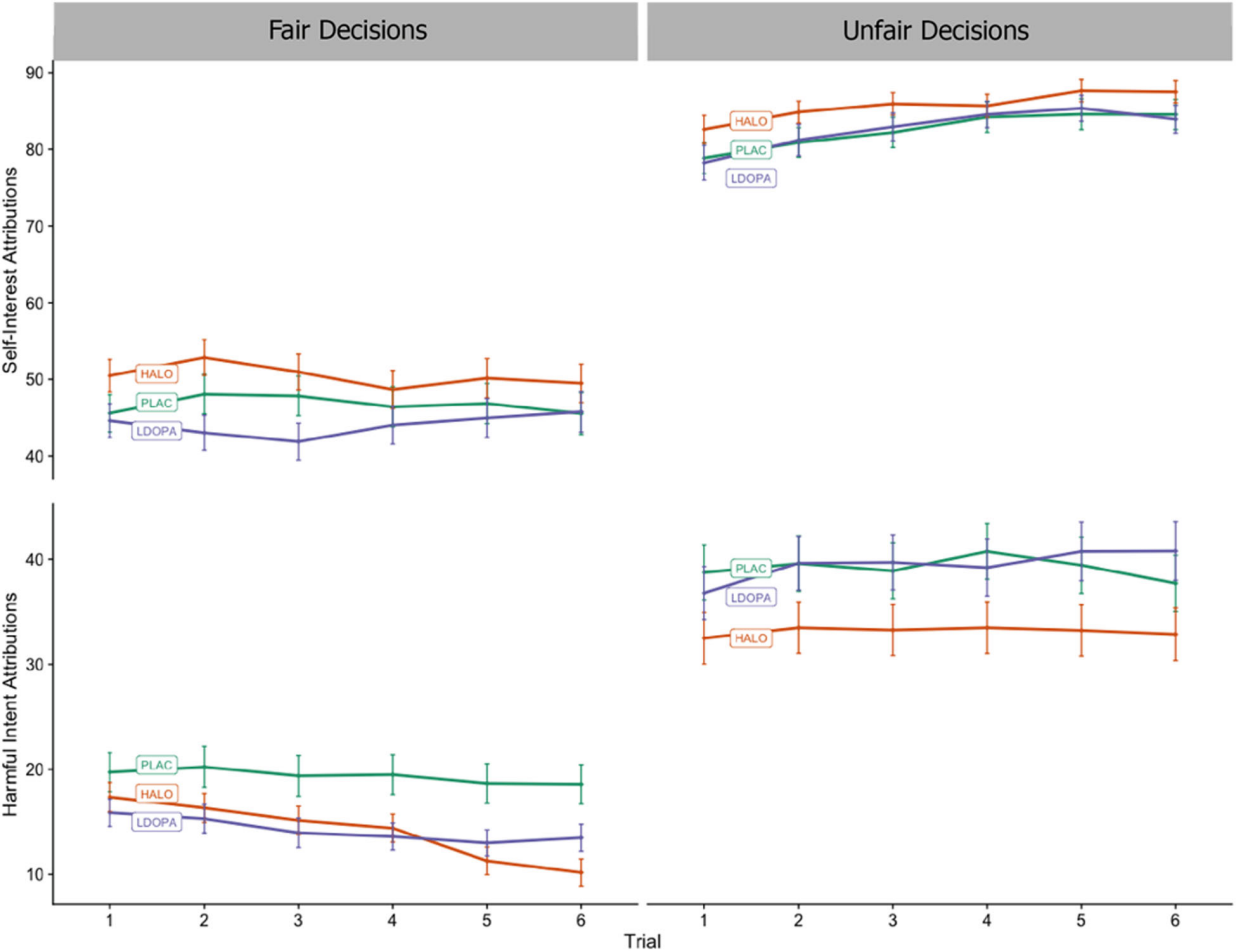
Trial by trial mean attributions of participants playing the multi-round Dictator Game for each drug condition, faceted by dictator type. Bars are the standard error of the mean. Partners were presented randomly to participants. For each trial, partners decided whether to split or keep £0.10; in unfair conditions, they always chose to keep it, and for fair conditions they always chose to split it. After each decision, participants attributed on a scale of 0–100 how much they thought their partner wanted to increase their own bonus (self-interest) and how much they thought their partner wanted to reduce their bonus (harmful intent). Relative to placebo, haloperidol demonstrates a reduction in harmful intent attributions across all dictator conditions. In fair conditions, haloperidol also demonstrates an increase in self-interest attributions. Relative to placebo, L-DOPA demonstrates a decrease of harmful intent attributions in fair conditions, and no difference compared to placebo in unfair conditions.

Figure 1 illustrates changes to harmful intent attributions and self-interest attributions for each trial, fair and unfair dictators, and drug condition.

We also conducted an additional analysis including drug condition, trait paranoia (GPTS Total), session number, dictator, and age, with ID and Trial as random effects.

For the main effect of drug condition across conditions, detailed in Table 3 (and illustrated in Appendix E), haloperidol reduced harmful intent attributions versus placebo, but increased self-interest attributions. L-DOPA showed no effects versus placebo (although we note marginal nonsignificance—the upper confidence interval at zero—for harmful intent attributions versus placebo with a small effect). Haloperidol decreased harmful intent but increased self-interest attributions versus L-DOPA. The unfairness of dictators and session number both increased harmful intent attributions (Table 3).

**Table 3:** Top model average for harmful intent attributions and self-interest attributions by drug, dictator, session number, paranoia, and age.

Parameter	Estimate	Standard error	95% CI		Relative importance
			Lower	Upper	
**Harmful Intent Attributions**					
* Intercept 1* | *2*	2.45	2.09	−1.64	6.54	
* Intercept 2* | *3*	3.91	2.09	−0.18	8.00	
* Intercept 3* | *4*	5.44	2.09	1.34	9.53	
* Intercept 4* | *5*	6.17	2.09	2.07	10.26	
Drug (haloperidol vs. placebo)	−0.61	0.09	−0.78	−0.44	1
Drug (L-DOPA vs. placebo)	−0.16	0.08	−0.33	0.00	1
Drug (L-DOPA vs haloperidol)	0.45	0.09	0.27	0.62	1
Dictator (fair < partially fair < unfair)	1.60	0.06	1.47	1.73	1
Paranoia (GPTS total)	0.08	0.29	−0.65	1.42	0.22
Paranoia (persecutory)	1.14	0.45	0.26	2.02	−
Session number (1 < 2 < 3)	0.19	0.06	0.07	0.31	1
Age	0.07	0.07	−0.01	0.22	0.68
**Self Interest Attributions**					
* Intercept 1* | *2*	−6.10	1.16	−8.38	−3.82	
* Intercept 2* | *3*	−5.11	1.16	−7.39	−2.83	
* Intercept 3* | *4*	−3.79	1.16	−6.07	−1.52	
* Intercept 4* | *5*	−2.28	1.16	−4.55	0.00	
Drug (haloperidol vs. placebo)	0.43	0.08	0.27	0.59	1
Drug (L-DOPA vs. placebo)	−0.10	0.08	−0.25	0.05	1
Drug (L-DOPA vs haloperidol)	−0.53	0.08	−0.69	−0.37	1
Dictator (fair < partially fair < unfair)	2.44	0.07	2.30	2.57	1
Paranoia (GPTS total)	−0.19	0.33	−0.84	0.46	0.3
Paranoia (persecutory)	−0.28	0.34	−1.09	0.10	0.57
Session number (1 < 2 < 3)	−0.34	0.06	−0.45	−0.23	1
Age	−0.10	0.04	−0.17	−0.03	1

ID and trial number were included as fixed effects. Model parameters: harmful intent/self interest attributions ~ drug + dictator + paranoia + session number + age + (1|ID) + (1|Trial). Models were selected and averaged based on their AICc criterion automatically in the “MuMIn” package. Beta estimates indicate the relationship between a term and harmful intent/self-interest attributions. GPTS total was included in the model, however we also report here the post-hoc statistics of the same model with the Persecutory Ideation subscale as a term instead. We also report the difference between L-DOPA, and haloperidol run in a separate model, as our main model only compared each active condition to placebo.

Total GPTS summed score did not have an effect on harmful intent nor self-interest attributions. However, previous work^40^ has instead used the Persecutory Ideation subscale of the GPTS as a term to assess paranoia, and so we also ran a model with this subscale as a term instead of the GPTS total. In this model, Persecutory Ideation was associated with an increase in harmful intent attribution but not self-interest attribution.

### Post-hoc analysis of changes in subjective mood and scepticism with attributions

We calculated whether there were any subjective effects of the drug on task performance by associating the change in the alertness subscale and tranquillity subscale^41^ between pre and postdose, and then additionally between drug and placebo conditions, with harmful intent and self-interest attributions. We found that mood changes were not associated with harmful intent or self-interest attributions (*p*’s > 0.05; see Supplementary Materials for plot). Likewise, we calculated whether participants’ beliefs about whether they were playing a real person influenced their harmful intent or self-interest attributions in any dictator condition under placebo, L-DOPA, or haloperidol. Participants were required to rate how much they believed they were playing against a real person on a scale of one to five, from ‘very sceptical’ to ‘totally believed the person was real’. At session one (first time being exposed to the game), 24 participants scored three or over, at session two, 20 participants score three or over, at session three, 21 participants scored three or over. We found that scepticism did not correlate with harmful intent or self-interest attributions for any drug or dictator condition (*p*’s > 0.05, see Supplementary Materials).

### Multi-round Dictator game prediction 2: dopamine manipulation will increase the rate at which high harmful intent attributions are reached, but not self-interest attributions

We conducted four preregistered analyses. There was no difference between L-DOPA and placebo (−0.37, 95% CI: −0.79, 0.05) or haloperidol and placebo (0.01, 95% CI: −0.45, 0.46) conditions for the trial where a harmful intent attribution score over 60 was triggered for unfair dictators. This was also true when running post-hoc analysis using the relative mean of the population for each dictator (haloperidol vs placebo: 0.28, 95% CI: −0.12, 0.69; L-DOPA vs placebo: 0.16, 95% CI: −0.24, 0.55).

Because so few people scored above 60 in any trial with fair partners before the final trial our model was unable to converge. We therefore ran a post-hoc analysis with the threshold set as the mean of the population (15.87). There was no difference between L-DOPA and placebo (0.01, 95% CI: −0.33, 0.32) or haloperidol and placebo (0.15, 95% CI: −0.19, 0.49) conditions for the trial where a harmful intent attribution score over 15.87 was triggered for fair dictators.

There was no difference between L-DOPA and placebo (0.01, 95% CI: −0.25, 0.26) or haloperidol and placebo (0.05, 95% CI: −0.20, 0.30) conditions for the trial where a self-interest attribution score over 60 was triggered for unfair dictators. There was no difference between L-DOPA and placebo (0.09, 95% CI: −0.26, 0.45) or haloperidol and placebo (0.00, 95% CI: −0.35, 0.35) conditions for the trial where a self-interest attribution score over 60 was triggered for fair dictators.

### Post-hoc analysis of change in score over time

To quantify whether dopamine manipulation adjusted the change in scores over trials for each dictator, we conducted a paired, within-subject analysis to assess the change in attributions between trial 1 and trial 6 under each drug condition.

Only haloperidol compared to placebo during unfair dictators demonstrated a reduction in harmful intent attributions between trials 1 and 6 (t^26^ = 3.68, *p* = < 0.001). There were no differences between drug conditions to changes in self-interest attributions between trial 1 and 6 (See Supplementary Materials for plot).

### Beliefs and values inventory

We administered the beliefs and values Inventory^31^ each study day after dosing. We predicted that manipulating dopamine would moderate the ratings of interest and self-relevance of paranormal beliefs.

We found that versus placebo, neither L-DOPA nor haloperidol changed the ratings of interest or self-relevance of paranormal statements. In exploratory analyses, we found that versus placebo, neither haloperidol nor L-DOPA changed any other dimensions of agreement, self-relevance or interest across themes of science, morality, politics, and religion (see Supplementary Materials).

## Discussion

We conducted a within-subjects, double-blind, randomised controlled study examining the effects of pharmacological manipulation of the dopamine system on attributions and beliefs in healthy participants. We found that modulating dopamine led to changes in social attributions relevant to paranoia but not the salience of beliefs across multiple themes. As predicted, and consistently across conditions, haloperidol reduced attributions of harmful intent versus placebo for opponents’ actions in a multi-round Dictator Game. Additionally, against predictions, haloperidol increased self-interest attributions against placebo. In contrast, L-DOPA showed no difference versus placebo for attributions of harmful intent, except in the fair condition where they were reduced. L-DOPA versus placebo reduced self-interest attributions in fair and unfair, but not partially fair conditions. Against predictions, we found that neither haloperidol nor L-DOPA influenced the rate at which attributions of increased harmful intent were made during serial interactions. As expected, Dictator fairness and pre-existing persecutory ideation both increased attributions of harmful intent, even when taking into account drug condition, replicating previous findings and providing evidence for the validity of the paradigm.

Our results were unlikely to be a general effect of sedation or reduction in social sensitivity, as haloperidol either had absent or condition-dependent opposite effects on measures of self-interest attribution for the same events. This suggests an important selective role for dopamine in attributions of harmful intent.

Current models of antipsychotic drug action propose that blockade of postsynaptic D2 receptors in striatal regions reduces aberrant salience thereby reducing psychotic symptoms^11^. While therapeutic effects in patients with psychosis are generally thought to take from days to weeks to establish, the present results suggest that D2 blockade is also associated with acute reductions in attributions of harmful intent in healthy individuals. This is consistent with proposals that D2 blockade produces acute effects on cognition^42^. While we cannot be certain of the brain regions underpinning our observed effects on social cognition, it is notable that striatal D2 receptors are associated with treatment effects of D2 antagonists in psychosis^34^, although dopaminergic agents provide important modulatory function in the prefrontal cortex.

While results from this study suggest that the dopamine system is likely to have a direct role in social attributions and particularly those relevant to paranoia, current mechanistic models of the role of dopamine in psychosis cite perceptual and cognitive factors that poorly account for its social content^43^. This may largely be because most experimental work on humans has focused on its role in general, rather than social cognition—for example, nonsocial reward^44^, risk and decision-making^45^, or nonsocial belief updating^46^. Given that we report evidence for the role of dopamine in appraisal of social threat, we suggest that dopamine modulates state representations of the social environment, much as nonsocial representations (e.g. stimulus reward relationships) are encoded by the interplay between the striatum and prefrontal cortex^47,48^. Indeed, it has been previously suggested that the integration of information in the striatum is critical for social interactions and relationships^49^. Specifically, we suggest that dopamine may modulate the representation of threat during social interactions, as social threat is an evolutionarily important focus of attention^50^. Evidence from mice, for example, suggests a specific subcortical dopaminergic circuit for environmental threat detection and avoidance^51^. The present findings in healthy participants indicate involvement of dopamine in attributions of harm; this may be relevant for attributions subsequently incorporated into normative or pathological beliefs^52^.

An unpredicted finding was that alongside decreasing attributions of harmful intent, haloperidol increased attributions of self-interest. This may indicate a more general involvement of dopamine in judgments about whether the intentions of social agents relate to the self or others. For example, reductions in attributions that behaviour is motivated by harmful intent may add inferential weight to alternative or competing appraisals of intention—such that disadvantageous behaviour is motivated by self-interest. However, L-DOPA was not associated with overall changes to attributions of self-interest indicating that the influence of dopamine manipulations on self-interest within this study is not symmetrical. This may be related to different mechanisms of action of the two compounds, with haloperidol blocking neurotransmission via postsynaptic dopamine D2 receptors and L-DOPA potentiating presynaptic dopamine synthesis.

We speculate that context dependent effects of L-DOPA (an effect limited to the fair condition) may reflect an interaction between the drug and the salience of others’ behaviours. We might have expected potentiating dopamine to increase paranoid attributions from the aberrant salience model^11^, although this model does not specify potential distinctions between paranoid attributions and those driven by presumed self-interest. Instead, we found that L-DOPA reduced attributions of both self-interest and harmful intent under fair conditions only. There are three key models of dopamine and behaviour within which we can frame these findings. While we do not have direct measures of dopamine activity, these models warrant consideration and may provide explanatory value (while not being mutually exclusive).

First, our findings may be explained by the sigmoidal model of dopamine, where dopamine increases a neuronal population’s response to strong inputs while diminishing it for weak inputs^53^. This fits with the L-DOPA findings for observed attributions of harmful intent and self-interest if we assume fair behaviour by a dictator provides a ‘weak’ input, and the unfair behaviour provides a stronger input (but still insufficient to be increased by L-DOPA). However, this model would predict increased attribution of harmful intent for haloperidol in the fair condition, whereas haloperidol decreased attributions of harmful intent.

The second model is a signal-to-noise account of dopaminergic modulation of neuronal activity and behaviour. Dopamine manipulations are known to affect signal-to-noise ratio, with L-DOPA predicted to both increase phasic signals while simultaneously increasing postsynaptic signal detection thresholds via increased tonic levels of dopamine^54,55^. Indeed, prior experimental evidence suggests that administration of L-DOPA in healthy, sceptical individuals reduces perceptual sensitivity, with the authors suggesting this was better explained by L-DOPA decreasing rather than increasing signal:noise ratio^29^. This model also requires the assumption that social behaviours produce different input signals at different levels of fairness. In this framework, under fair conditions, the input signal may be too weak to overcome a higher set threshold for attributing intentions to another agents’ behaviour (fitting the observed reduction in self-interest and harmful intent). By contrast, unfair conditions are more salient and therefore readily cross a higher set threshold for attributing intentions that would otherwise be made without L-DOPA (in the placebo condition). Conversely, there is a reduction in overall signalling via postsynaptic D2 receptor blockade with haloperidol. This may explain the reduction in harmful intent attributions, although does not easily explain the increase in self-interest attributions. Changes in attributions of self-interest may be better understood by the reductions in attributions of harmful intent adding inferential weight to the alternative/competing appraisals of intention.

Finally, the effect of dopamine manipulations are often interpreted in an ‘inverted-U’ model, whereby increases or decreases in dopamine outside an optimal signalling window lead to a decrease in behavioural response. Nonlinear effects of dopamine modulation have been reported in decision-making^56^, working memory^57^, sensation-seeking^58^, and lexical decision tasks (Krummenacher et al.^29^). The data presented here suggest this may also be extended to social cognitive function in general and attributions of harmful intent and self-interest, specifically. Within this inverted-U model, haloperidol reduced attributions of harmful intent by reducing overall postsynaptic dopamine transmission via D2 receptors to the left of the optimum. At the same time self-interest attributions increase suggesting a separate inverted-U model for different attributions. Increased DA transmission can disrupt behaviour^57^, and for this model to fit with our L-DOPA findings would also require the added assumption that different task conditions likely have different optimal dopamine levels^59^. Thus, L-DOPA may raise dopamine release above optimal levels in fair conditions, but not potentiate dopamine enough outside optimal levels in partially fair or unfair conditions to make a difference to attributions—where a different optimal level and inverted-U model may apply.

Another possibility is the lack of significant increase in harmful intent attributions with the administration of L-DOPA overall may be attributed to the dose being insufficient. However, we find this less likely, as L-DOPA affected other aspects of the task and prior studies using L-DOPA at the same dose showed modulation of decision-making processes, including those made within a social context^26,45^.

Other factors may explain the findings we observed for L-DOPA. The lack of a significant increase in harmful intent attributions under unfair conditions with L-DOPA may reflect a ceiling effect in participants with low levels of trait paranoia. It may also be that persistent increases in presynaptic dopamine release over time, coupled with sustained environmental stresses (including threatening behaviours), leads to sustained increases in attributions of harmful intent as the basis for paranoid beliefs. Paranoid states produced by drugs such as amphetamine, typically happen at high doses or after persistent use (Lecomte et al.^19^), and it has been suggested that this also occurs with the use of L-DOPA in Parkinson’s disease^60^.

We also did not find an effect of either dopamine manipulation on the salience of paranormal beliefs—selected as an analogue of delusional ideation—and assessed using the BVI^31^ alongside beliefs about politics, morality, religion, and science. Aberrant salience models of psychosis^18^ suggest that delusional beliefs are the outcome of sustained disruptions to striatal dopamine. Consequently, it may be that relatively brief changes to dopamine transmission are not sufficient to produce detectable changes in the salience of propositional beliefs, for which attitudes tend be more stable^31^.

### Limitations

We use a relatively short social inference task that may preclude assessment of behaviours over a longer period of time. Previous nonsocial tasks (e.g. ref. ^44^) and more recent studies with iterative social interactions (e.g. ref. ^61^), have used comparatively longer trial designs, some in excess of 100 trials. It could be argued that some dynamics of social inference may not be evident without viewing more decisions. It remains an open question as to whether our observed drug effects would be sustained given longer social interactions, or whether we may observe sensitised responses. Also, we only used one dose of each compound and additional doses could potentially reveal a nonlinear dose-response profile. There are some obvious sampling biases in our design, namely that we use all males, have a relatively small sample, and have recruited healthy individuals that happened to see our advertisement from the local community.

## Conclusions

We conducted a double-blind, within-subjects randomised controlled study in healthy individuals to test the effect of dopamine modulation on social inferences related to paranoia. We report evidence for the role of dopamine in the attribution of others’ intent to harm. Importantly, our findings were not attributable to subjective mood, beliefs in general, nor scepticism about whether participants were playing real partners. These findings are consistent with imaging and physiological evidence^49^, and evolutionary accounts^50^, that identify a key role for dopamine in social inference. Future research should aim to use live, social process-oriented tasks in combination with imaging and pharmacology to better understand the role of dopamine in social attributions and its interaction with psychosocial factors (such as social stress) which are known to increase risk for psychosis.

## Methods

### Ethics and recruitment

This study was approved by KCL ethics board (HR-16/17-0603). All data were collected between August 2018 and August 2019. Participants were recruited through adverts in the local area, adverts on social media, in addition to adverts circulated via internal emails.

Eighty-six participants were preliminarily phone screened. 35 participants were given a full medical screen. Thirty healthy males were recruited to take part in the full procedure. Inclusion criteria were that participants were healthy males, between the ages of 18 and 55. Participants were excluded if they had any evidence or history of clinically significant medical or psychiatric illness; if their use of prescription or non-prescription drugs was deemed unsuitable by the medical team; if they had any condition that may have inhibited drug absorption (e.g. gastrectomy), a history of harmful alcohol or drug use determined by clinical interview, use of tobacco or nicotine containing products in excess of the equivalent of five cigarettes per day, a positive urine drug screen, or were unwilling or unable to comply with the lifestyle guidelines. Participants were excluded who, in the opinion of the medical team and investigator, had any medical or psychological condition, or social circumstance, which would impair their ability to participate reliably in the study, or who may increase the risk to themselves or others by participating. Some of these criteria were determined through telephone check for nonsensitive information (age, gender, general understanding of the study, and overall health) before their full screening visit.

### Procedure

Participants attended 4 days in total at the Centre for Neuroimaging Sciences in Denmark Hill. The first day consisted of the full medical screen that lasted approximately an hour, in addition to providing participants with a consent form to review. If participants chose to consent to take part the screening day commenced. Participants were excluded if they were currently unwell (e.g. a cold), or if they had begun any new medication that was deemed unsuitable by medical staff. Participants underwent urinalysis, a drug screen (testing for Amphetamines, Barbiturates, Benzodiazepines, Cocaine, THC, Methadone, Methamphetamine, Opiates, Phencyclidine, and Tricyclic Antidepressants); participants were rejected if they tested positive for any of the above. Participants were also weighed and measured, and any participants with a BMI over 30 were excluded.

Electrocardiograms were taken, and participants excluded if parameters were exceeded (QTc: 330–430; PR: 120–210; QT: 270–470; QRS: < 120; Heart Rate: 40–90 bpm). Additionally, blood pressure was taken, with acceptable mmHg within 90–140 (systolic) and 40–90 (diastolic) when supine and after 2 min of standing.

A neurological assessment was made by the medical team, testing for tremor, nystagmus, pupillary reactivity, reflex test, finger-nose test, Romberg’s sign, gait, shoulder girdle strength, upper extremities strength, lower extremities strength, and myoclonic jerks. General appearance, dermatological signs, respiratory signs, cardiovascular health, abdominal signs, extremities, and musculoskeletal signs were all assessed, and participants included if normal.

Participants were given a full psychiatric exam by the medical team and excluded if any clinically significant signs or symptoms were reported, either currently or historically.

Participants then completed the OCEAN personality questionnaire^37^, Brief OLIFE^38^, Green Paranoid Thoughts Scale^36^, and Bond and Lader mood rating scale^39^.

At least 7 days later participants were then invited back for the first study session if they had satisfactorily passed the assessment day. Participants were paid £20 if they failed the screening day. Each study day was spaced by at least 7 days, but no more than two months. Each study day was identical in procedure. Participants were requested to abstain from alcohol and caffeine at least 24 h before the study day. Study days began with a similar screening procedure to the screening day. ECGs, blood pressure, urinalysis, drug screening, neurological, and physical checks were all completed upon arrival. Participants were also asked to complete the Bond and Lader mood rating scale prior to initial dosing.

Participants were initially dosed in the morning between 9.30 and 10.30 a.m. Participants were randomly (in a Williams Square design; ref. ^62^) administered 3 mg of haloperidol in two capsules or placebo in two capsules, and 10 mg of Domperidone or placebo in one capsule (three capsules total).

After an hour and a half, participants were dosed a second time. This would randomly be assigned as 150 mg of co-beneldopa or placebo in two capsules. Participants never took both haloperidol and co-beneldopa on the same day. Participants were also provided with a light lunch following the second dosing session. Participants only drank water throughout the entirety of the day.

After an hour and a half, participants were dosed a second time. They would randomly be assigned 150 mg of co-beneldopa or placebo in two capsules. Participants never took both haloperidol and co-beneldopa on the same day. Participants were also provided with a light lunch following the second dosing session. Participants only drank water throughout the entirety of the day.

In sum, participants were either given Haloperidol (3 mg) + Placebo, Domperidone (10 mg) + L-DOPA (150 mg), or Placebo + Placebo.

Participants were then discharged. Discharge consisted of an ECG, blood pressure assessment, neurological, and physical exam by the medical team. If participants required a taxi they were provided with one. If participants reported any adverse events these were recorded.

### The multi-round dictator game

We developed a within-subjects, multi-trial modification on the Dictator game design used in previous studies to assess paranoia^33^. Each participant played six trials against three different types of dictator. In each trial, participants were told that they have been endowed with a total of £0.10 and their partner (the dictator) had the choice to take half (£0.05) or all (£0.10) the money from the participant. Dictator decisions were one of three types: either to always take half of the money, have a 50:50 chance to take half or all of the money, or always take all of the money, labelled as fair, partially fair, and unfair, respectively. The order that participants were matched with dictators was randomised. Each dictator had a corresponding cartoon avatar with a neutral expression to support the perception that each of the six trials was with the same partner.

After each trial, participants were asked to rate on a scale of 1–100 (initialised at 50) to what degree they believed that the dictator was motivated (a) by a desire to earn more (self-Interest), and (b) by a desire to reduce their bonus in the trial (harmful intent). Following each block of six trials participants were asked to rate the character of the dictator overall by scoring intention again on both scales. Therefore, participants judged their perceived intention of the dictator on both a trial-by-trial and partner level.

After making all 42 attributions (two trial attributions for each of the six trials over three partners, plus three additional overall attributions for each partner), participants were put in the role of the dictator for six trials—whether to make a fair or unfair split of £0.10. Participants were first asked to choose an avatar from nine different cartoon faces before deciding on their six different splits. These dictator decisions were not used for analysis but were collected in the first phase of the game to match subsequent participants with decisions from real partners.

The modification to the original dictator game design allowed us to track how partner behaviour, order of partner, and whether attributions were highly variable or consistent as pre-existing paranoia changed. All participants were paid for their completion of the GPTS, regardless of follow up. Participants were paid a baseline payment for their completion.

### Analysis

We used an information-theoretic approach for all analyses unless otherwise stated. Following Barnby et al.^33^, we analysed the data using multi-model selection with model averaging^63,64^. The Akaike information criterion, corrected for small sample sizes (AICc), was used to evaluate models, with lower AICc values indicating a better fit^64^. The best models are those with the lowest AICc value. To adjust for the intrinsic uncertainty over which model is the true ‘best’ model, we averaged over the models in the top model set to generate model-averaged effect sizes and confidence intervals^63^. In addition, parameter estimates, and confidence intervals are provided with the full global model to robustly report a variable’s effect in a model^65^. This used package “MuMIn”^66^. All analyses were conducted in R^67^. All visualisations were generated using the package ‘ggplot2’^68^.

In our models, all baseline continuous scale scores were centred and scaled to produce Z values. All model statistics reported are beta coefficients.

Scores of harmful intention attributions and self-interest for each dictator were taken over six trials for analysis. These were used for cumulative link mixed models (clmm; ref. ^69^). In our confirmatory analysis, for each dictator harmful intent or self-interest attributions were set as our dependent variables and ID set as a random term:

Formula: Value (Ordinal) ~ Drug + (1|ID)

In our exploratory analysis, harmful intent and self-interest attributions were set as our dependent variable. Paranoia (GPTS and Persecutory subscale), dictator behaviour (fair, unfair, and partially fair), age, drug (Placebo/haloperidol/L-DOPA) were set as our explanatory terms with ID and Trial set as random terms.

Formula: Value (Ordinal) ~ Drug + Paranoia + Dictator + Session Number + Age + (1|ID) + (1|Trial)

For our second prediction, participants that scored above 60 were considered to have scored high harmful intent attributions. Both harmful intent and self-interest scores participants were set a value of 6 if they had scored 60 in their first trial, 5 if they had scored over 60 by their second trial, 4 if they had scored 60 by their third trial, and so on. All trials following the threshold being reached were coded as 0. Participants not reaching the threshold for any trial were coded 0 across all trials. Both unfair and fair dictator behaviour were analysed with two cumulative link mixed models (clmm) each, one for harm-intent and one for self-interest.

Formula: Trial (where score > 60 triggered) ~ Drug + (1|ID)

For attribution changes between trials one and six for each dictator and attribution type we used the R package “ggstatsplot”^70^.

## Supplementary information

Supplementary Material

## Data Availability

All data can be found on the Open Science Framework: https://osf.io/mr63j/.

## Code availability

All analysis code can be found on the Open Science Framework: https://osf.io/mr63j/.
