# Supplementary material for: Dopamine manipulations modulate paranoid social inferences in healthy people
Source: Transl Psychiatry. 2020 Jul 5;10:214. doi: 10.1038/s41398-020-00912-4 (PMC7335741; doi:10.1038/s41398-020-00912-4)

**Supplementary Material**

*Appendix A. The Beliefs and Values Inventory across conditions.* Aggregate scores of participants that answered the Beliefs and Values Inventory in each drug condition. Scores are divided by themes (facets) and dimensions (x-axis). Dots resemble the mean. Bars represent the standard error of the mean.

**
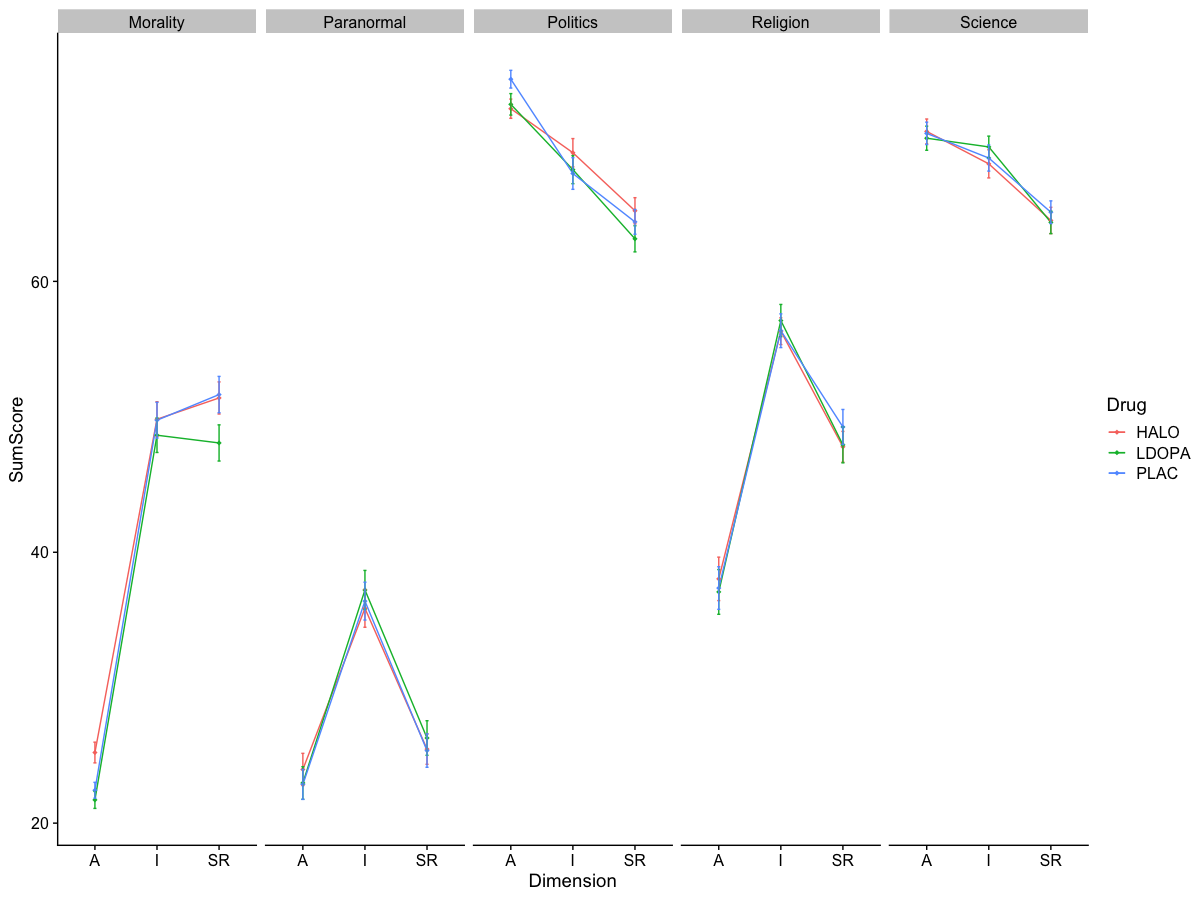
**

*Appendix B. Changes in subjective mood between drug and placebo conditions following dosing.* Aggregated association of scores on the Alertness and Tranquil subscales of the Bond and Lader Visual Analogue Scale between LDOPA and Placebo, and haloperidol and Placebo conditions, with Harmful Intent and Self Interest attributions. Grey shading represents the standard error of the mean.

**
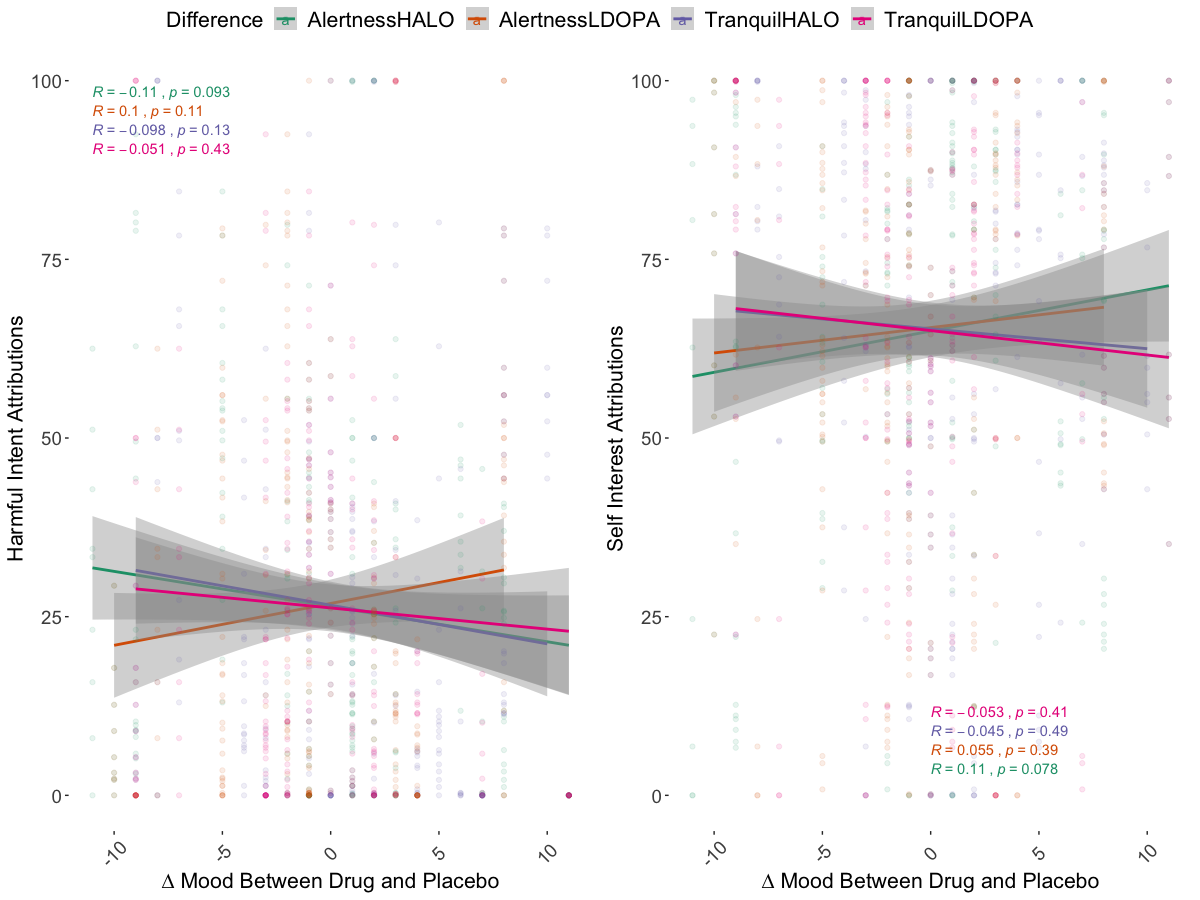
**

*Appendix C. Association between scepticism scores and attributions across dictator conditions.* Grey shading represents the standard error of the mean.

**
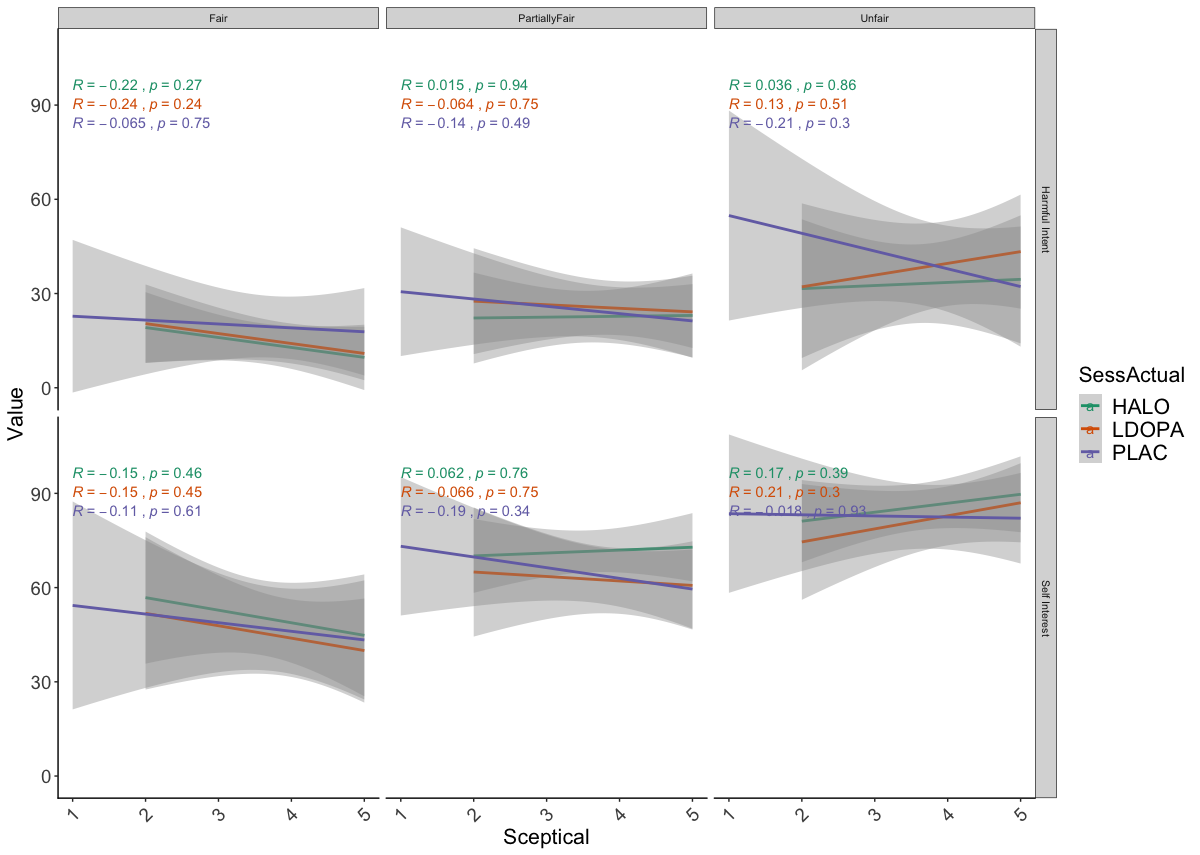
**

*Appendix D. Change in attribution scores between trial 1 and 6 for unfair and fair partners for each drug condition.*

**
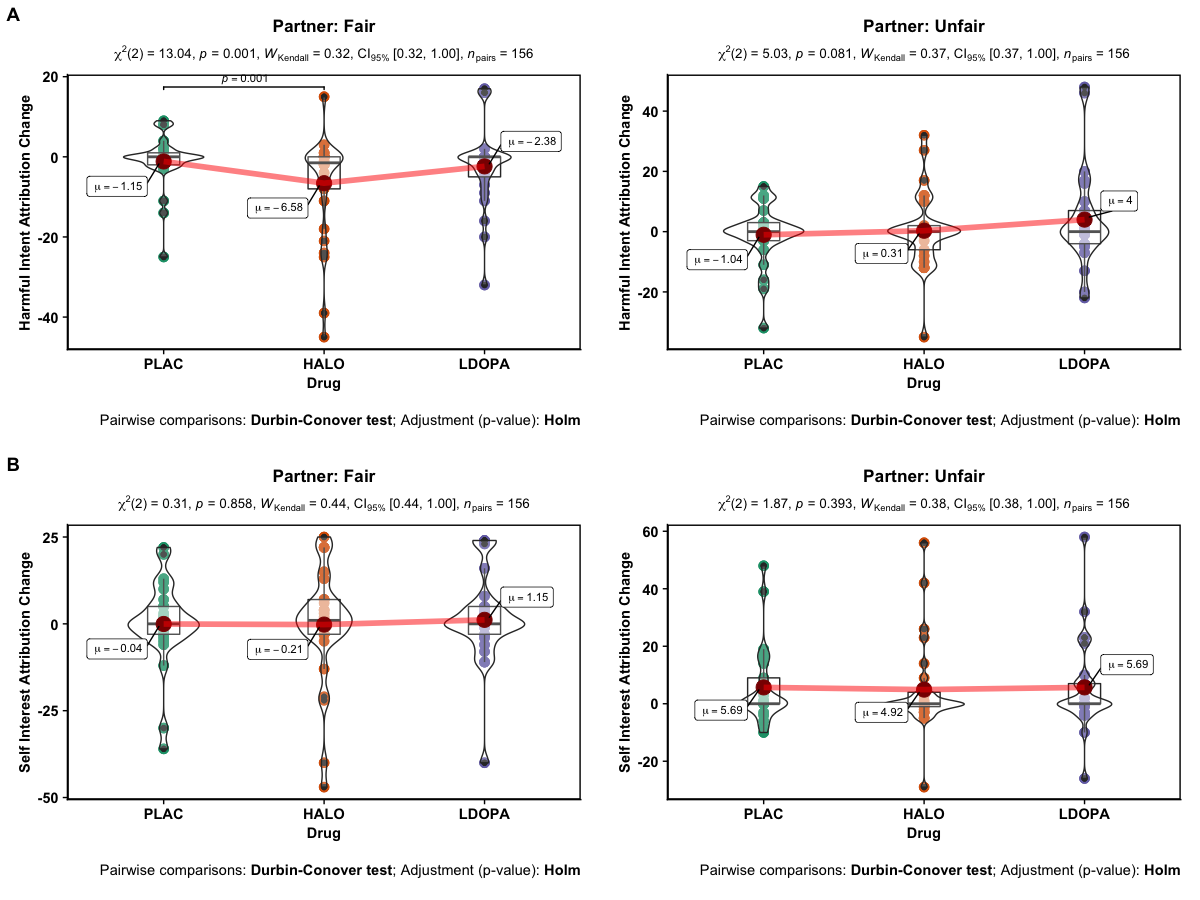
**

*Appendix E. Mean and Standard Deviation of Harmful Intent Attributions and Self Interest Attribution for each drug condition, collapsed across social conditions and trials (unfair, fair, and partially fair).*


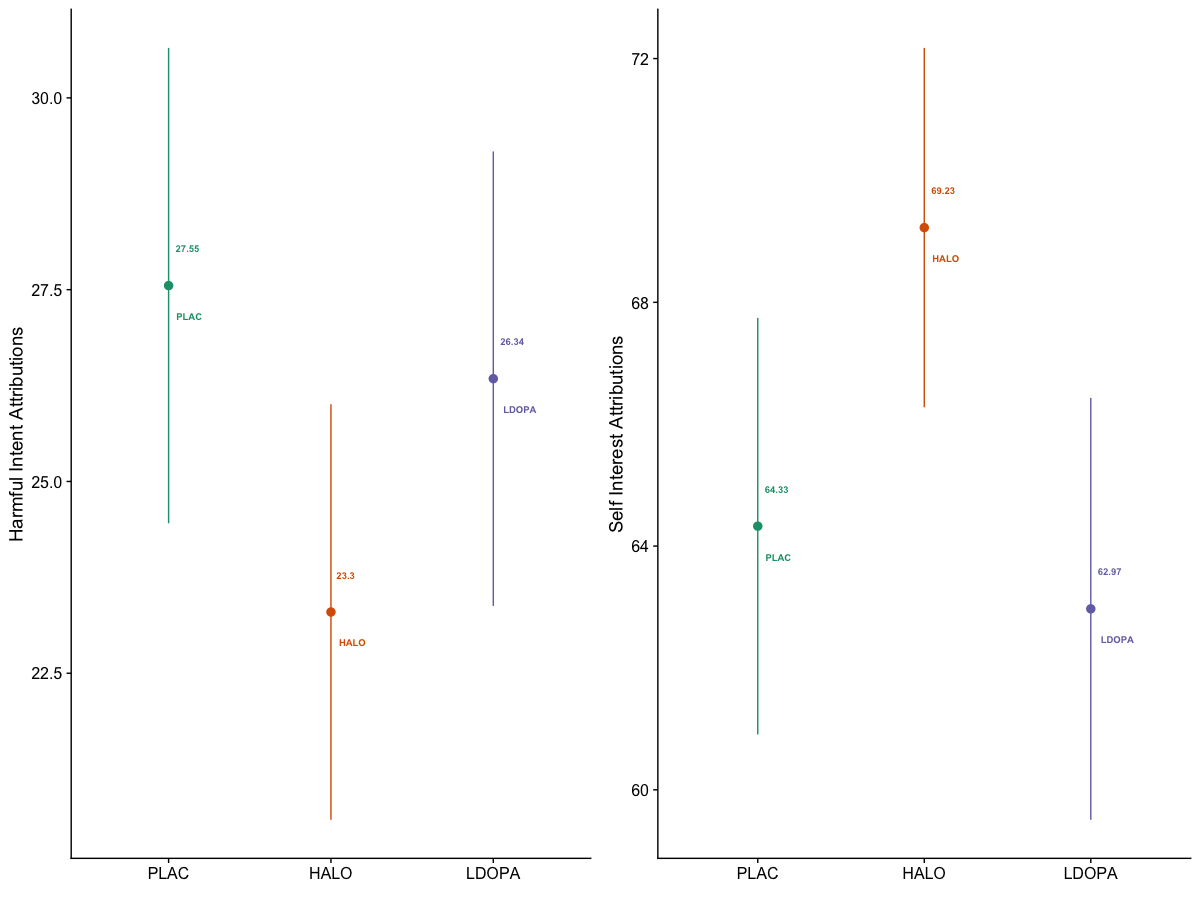

Supplement: Supplementary file 1 — Supplementary Material [file 41398_2020_912_MOESM1_ESM.docx]
